# Functionalization of *Tenebrio molitor* with Olive Mill Wastewater: Growth, Antioxidant Activity, and Metabolomic Insights

**DOI:** 10.3390/ijms27073201

**Published:** 2026-04-01

**Authors:** Annalaura Brai, Giuseppe Galeone, Alessio Maccianti, Federica Poggialini, Chiara Vagaggini, Elena Dreassi

**Affiliations:** Department of Biotechnology, Chemistry and Pharmacy, University of Siena, Via A. Moro, 53100 Siena, Italyfederic.poggialini@unisi.it (F.P.); chiara.vagaggini@student.unisi.it (C.V.); elena.dreassi@unisi.it (E.D.)

**Keywords:** circular economy, edible insects, agri-food by-products, waste valorization, NMR metabolomics, bioactive compounds

## Abstract

Olive mill wastewaters (OWWs) are a phenol-rich by-product of olive oil production, associated with high disposal costs and significant environmental impact. This study evaluated the effects of OWW supplementation on *Tenebrio molitor* larvae (TML) reared on standard cereal-based substrates. Larval survival was not affected, but average body weight was significantly reduced in all OWW-treated groups, indicating a measurable impact on growth. Metabolomic profiling revealed alterations in amino acid, carbohydrate, and organic acid contents, including reductions in branched-chain and aromatic amino acids, trehalose, and Krebs cycle intermediates, particularly in groups with the strongest growth reduction. Lipid analysis showed stable saturated fatty acids, a shift from polyunsaturated to monounsaturated fatty acids, and an increase in total fat in the most affected groups. Despite the lack of enhancement in polyphenol accumulation or antioxidant activity, multivariate analysis highlighted distinct metabolic signatures between OWW-treated and control larvae, with sucrose, lactate, and fumarate identified as key discriminant metabolites. These findings demonstrate that OWW, a valorized olive oil by-product, can modulate growth and metabolism in TML, opening new perspectives for its application in innovative weight management strategies.

## 1. Introduction

Olive mill wastewater (OWW) is a by-product of olive oil production characterized by high volumes and a complex chemical composition. In the Mediterranean region, olive oil production generates between 10 and 30 million cubic meters of OWW per year, depending on olive variety, extraction method, and climatic conditions [1,2]. These effluents exhibit a high organic load, with chemical oxygen demand (COD) values ranging from 40 to 150 g/L, making them considerably more polluting than typical municipal wastewater [3]. Due to their phytotoxic and antimicrobial properties, their discharge is strictly regulated, and untreated disposal can lead to severe soil and water contamination [4]. Consequently, research has focused on developing integrated treatment strategies that combine physical, chemical, and biological processes to achieve efficient pollutant removal while recovering valuable compounds from the wastewater [5]. OMW treatment typically combines physical (sedimentation, centrifugation, membrane filtration), chemical (coagulation, advanced oxidation), and biological processes. Physical methods reduce solids and concentrate polyphenols, while chemical and biological treatments degrade phenolics and organic matter, with some biological approaches also producing value-added products like biogas [6,7,8,9]. Despite these technological advances, OMW treatment remains costly, with estimated expenses ranging from €18,000 to €27,000 per 1000 m^3^ depending on the method employed, posing a particular challenge for small- and medium-sized mills [10,11]. These challenges have prompted a growing interest in valorization strategies that convert OWW from an environmental burden into a resource for other production systems, aligning with principles of the circular economy [12].

One promising approach is the use of agro-industrial by-products as feed substrates for *Tenebrio molitor* larvae (TML). In 2021, the European Commission authorized dried TML as a Novel Food under Regulation (EU) 2015/2283, following EFSA’s positive safety opinion, marking them among the first insects approved for human consumption in the EU [13,14].

From a nutritional perspective, TML provide complete digestible proteins and a lipid fraction dominated by mono- and polyunsaturated fatty acids, including essential fatty acids, making them comparable to conventional animal protein sources [15,16]. Their remarkable ability to convert low-value organic substrates into nutrient-dense biomass makes them ideal candidates for integrating low-cost and waste-derived feed ingredients within a sustainable production framework [17]. Indeed, numerous studies have demonstrated that supplementing TML diets with agricultural by-products such as tomato peels and seeds, fallen plant leaves, or residues from cereal processing increases antioxidant capacity and favorably modifies the fatty acid profile of the larvae [18,19]. However, certain plant essential oils (e.g., oregano, thyme, garlic, caraway) have been reported to exert repellent or, in many cases, strong insecticidal effects. Secondary metabolites can influence insect survival, behavior, and development through multiple modes of action, highlighting the need for careful selection of dietary components [20,21,22,23].

Insect performance is also strongly influenced by diet composition. This relationship is context-dependent, as several studies have shown that diets excessively rich in protein can, in some cases, lead to increased mortality and reduced overall production performance. Consequently, insect performance appears to be more strongly influenced by a balanced nutrient composition, with particular emphasis on the carbohydrate-to-protein ratio, rather than by high protein content alone [24,25,26,27,28]. Additionally, the TML ability to accumulate bioactive compounds from the feed suggests that diet formulation can be used strategically to enhance functional properties. Recent research has also explored the bioactive potential of TML beyond basic nutrition, identifying peptides and metabolites with anti-hypertensive, antioxidant, and neuroprotective activities in vitro and in vivo, further underscoring the species’ value not only as a food ingredient but as a source of functional molecules [15,29,30].

Building on these insights, we investigate the potential use of OWW as a feed supplement for TML. By incorporating OWW into commonly used larval substrates (e.g., oat bran, wheat bran, rolled oats), this work aims to evaluate whether the high content of nutrients and antioxidant compounds inherent to OWW can be transferred to the larvae, thereby enhancing both their growth performance and nutraceutical value. Through this research, the study proposes a valorization pathway that integrates waste management, sustainable insect rearing, and the production of nutrient-rich food ingredients in alignment with circular economy principles.

## 2. Results and Discussion

### 2.1. Analysis of OWW

#### 2.1.1. Physicochemical Parameters of Olive Mill Wastewaters (OWWs)

Olive mill wastewater (OWW) samples have been analyzed for their basic physico-chemical parameters in particular pH, Total Solids (TS), and Total Suspended Solids (TSS). As shown in Table S1, the physicochemical characterization of the three olive mill wastewater samples (OWW1–OWW3), collected during the centrifugation phase of oil extraction, revealed the typical features of fresh olive mill wastewater. The pH values ranged from 5.01 to 6.18, confirming the slightly acidic nature of OWW. OWW1 showed the highest pH (6.18), whereas OWW2 and OWW3 were more acidic (5.01 and 5.24, respectively). These values are consistent with those commonly reported for olive mill wastewater, whose acidity is mainly due to the presence of organic acids, phenolic compounds, and residual fermentation products formed during olive processing [31].

Marked differences were observed in total solids (TS). OWW1 exhibited 75.82 ± 5.59 g/L, while OWW2 and OWW3 showed significantly higher concentrations (101.08 ± 5.44 and 107.95 ± 2.28 g/L, respectively). These values are in line with published data for centrifugation-derived OWW, where TS concentrations frequently range from 50 to over 100 g/L depending on olive cultivar, maturity stage, and extraction technology [29,30]. The high TS content reflects the substantial load of dissolved and suspended organic matter, including sugars, organic acids, polyphenols, proteins, and residual oil fractions. From an environmental perspective, such elevated solid concentrations contribute to the high chemical oxygen demand (COD) typically associated with OWW and explain the need for appropriate treatment before disposal [32].

A similar trend was observed for total suspended solids (TSS). OWW2 and OWW3 showed the highest particulate load (10.85 ± 0.66 and 9.90 ± 1.41 g/L, respectively), while OWW1 presented a lower but still considerable value (5.26 ± 1.66 g/L). The relatively high TSS values confirm that wastewaters collected directly during centrifugation contain a substantial fraction of suspended particles, including cell wall fragments, colloidal material, and phenolic-rich solids. Literature reports TSS values in centrifugation OWW typically between 5 and 15 g/L, which is consistent with the present findings [31,32]. The combination of high TS and TSS highlights the strong pollution potential of these effluents but also indicates the presence of bioactive compounds that may be valorised in a circular economy perspective.

Nitrogen content, expressed as percentage on dried samples, showed comparable values in OWW1 and OWW2 (2.21 ± 0.19%), whereas OWW3 exhibited a markedly lower concentration (0.11 ± 0.03%). The values observed in OWW1 and OWW2 fall within the range commonly reported for olive mill residues, where nitrogen mainly derives from proteins, peptides, and other nitrogenous compounds released from olive pulp [33]. The substantially lower nitrogen content of OWW3 suggests compositional variability potentially related to differences in olive cultivar, agronomic conditions, or processing parameters. Such variability is well documented in the literature and reflects the strong influence of raw material characteristics on OWW composition [31,33].

Residual fat content also differed among samples. OWW1 showed the highest value (2.62 ± 0.27%), OWW3 an intermediate level (1.11 ± 0.06%), and OWW2 the lowest (0.59 ± 0.02%). These differences are likely associated with the efficiency of oil separation during centrifugation and the specific technological settings of each mill. Published studies indicate that residual oil in OWW can vary widely, depending on extraction yield and decanter performance [34,35]. The presence of residual lipids not only contributes to the overall organic load but may also represent a recoverable fraction of interest for valorisation strategies.

Overall, the data confirm that OWW1–OWW3, all collected during the centrifugation phase, exhibit physicochemical characteristics typical of high-organic-load olive mill wastewater. The variability observed among samples reflects differences in processing conditions and raw material properties, in agreement with previously published studies [31,33]. These results underline both the environmental burden associated with untreated OWW and the potential for recovery of valuable components within an integrated waste management framework.

#### 2.1.2. Antioxidant Activity of Olive Mill Wastewaters (OWWs)

The total phenolic content (TPC) and antioxidant activity of the olive mill wastewaters (OWW1–OWW3) are reported in Table S1. TPC varied significantly among the samples, with OWW2 showing the highest value (9.94 ± 1.12 g GAE/L), followed by OWW3 (3.46 ± 0.49 g GAE/L) and OWW1 (1.05 ± 0.15 g GAE/L). Radical scavenging assays (DPPH and ABTS) showed a similar trend. OWW2 and OWW3 exhibited strong antioxidant activity, while OWW1 showed markedly lower activity. These results suggest that phenolic compounds are the main contributors to the antioxidant capacity of OWW. The observed variation among samples is consistent with the literature, where OWW collected directly from centrifugation contains higher concentrations of bioactive phenolics, such as hydroxytyrosol, tyrosol, and oleuropein aglycone, compared to wash or diluted waters [36,37,38]. The correlation between DPPH and ABTS assays confirms the strong radical scavenging potential of phenolic-rich OWW. Overall, OWW2 and OWW3 represent promising sources of natural antioxidants, suitable for valorization in nutraceutical, cosmetic, or functional applications. The data emphasize the importance of collection timing and processing conditions in maximizing phenolic content and bioactivity.

#### 2.1.3. Fatty Acid Composition of Olive Mill Wastewaters (OWWs)

As reported in Table S2, the fatty acid (FA) profile of OWW1–OWW3 was dominated by monounsaturated fatty acids (MUFA), followed by saturated (SFA) and polyunsaturated fatty acids (PUFA), reflecting the typical lipid composition of olive-derived matrices [31]. Total SFA ranged from 14.68% (OWW2) to 15.49% (OWW3). Palmitic acid (C16:0) was the main saturated fatty acid (12.07–13.07%), followed by stearic acid (C18:0) (1.94–2.06%). These values are consistent with those reported for virgin olive oil and olive by-products, where SFA generally account for 12–20% of total fatty acids [31,34].

MUFA represented the predominant fraction (76.45–80.16%), largely due to oleic acid (C18:1 Δ9), which ranged from 72.04% (OWW1) to 79.06% (OWW3). This is in agreement with literature data describing olive oil as a MUFA-rich matrix, typically containing 70–80% oleic acid depending on cultivar and geographical origin [39]. Minor MUFA (C16:1 and C17:1 isomers) were detected in lower proportions, with some variability among samples. PUFA accounted for 6.79–7.56% of total fatty acids. Linoleic acid (C18:2 Δ9,12) was the main PUFA (5.98–6.41%), followed by small amounts of C18:3. These values fall within the expected range reported for olive oil derivatives (5–15% linoleic acid) [31,39].

The observed fatty acid composition in OWW aligns with that of olive oil, consistent with the presence of residual lipids derived from fractions not entirely captured during centrifugation. The high MUFA content, particularly oleic acid, suggests potential for lipid recovery and valorization in nutraceutical, cosmetic, or feed applications.

### 2.2. Formulation and Analysis of Diets

For the feeding trials, three reference diets—wheat bran (WB), oat bran (OB), and whole oat flakes (OF)—were selected based on their similar composition and suitability for TML rearing (Table S3). Each diet was enriched with olive mill wastewater (OWW) at a fixed ratio, resulting in three enriched feeds of OB+, WB+ and OF+, respectively.

**Figure 1:**
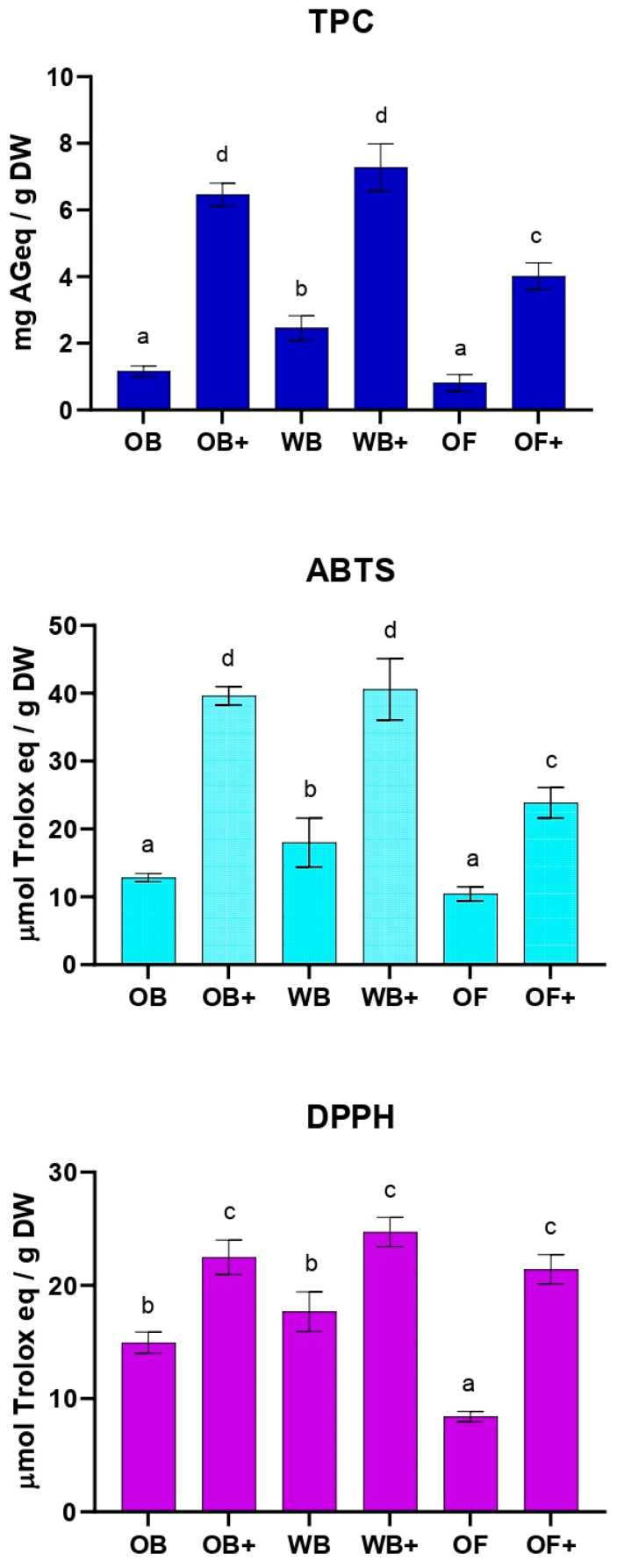
Total phenolic content (TPC) and antioxidant activity measured by ABTS and DPPH assays in oat bran (OB), wheat bran (WB), whole oat flakes (OF), and OWW-enriched diets (OB+, WB+, OF+). TPC was expressed as milligrams of gallic acid equivalents per gram of dry weight (mg GAE/g DW), antioxidant capacity was expressed as µmol of Trolox equivalents per gram of dry weight (µmol TE/g DW). Results are expressed as mean ± SD of at least three independent experiments. Statistical analysis was performed using one-way ANOVA followed by Tukey’s post hoc test. Values labeled with different letters (a, b, c, d) are significantly different (*p* < 0.05).

The diets were extracted and analyzed for their polyphenol content and antioxidant activity. As shown in Figure 1, total phenolic content (TPC) and total antioxidant activity (TAC) measured by ABTS and DPPH assays exhibited statistically significant differences. In all OWW-enriched diets (OB+, WB+, and OF+), the observed increases in antioxidant compounds and corresponding antioxidant activities reflect the direct contribution of the phenolic-rich OWW, as expected from the supplementation.

### 2.3. Effects on Tenebrio molitor Larvae

For the rearing trials, each group of TML received the control diets (OB, WB, OF) or the enriched diets (OB+, WB+ and OF+). Larval survival and body weight were monitored at 15, 30, and 45 days. The growth performance of TML, expressed as mean larval weight over the rearing period, is reported in Table 1 for the reference diets (OB, WB, OF) and their OWW-enriched counterparts (OB+, WB+, OF+). Mortality was minimal, with no more than two larvae lost per tray, and the highest growth rates were consistently observed on the wheat bran diet. Two-way ANOVA revealed that both time (F(3,48) = 661.4, *p* < 0.0001; 73.9% of total variation) and diet (F(5,48) = 65.33, *p* < 0.0001; 12.2% of total variation) significantly affected larval weight. The time × diet interaction was also significant (F(15,48) = 21.64, *p* < 0.0001; 12.1% of total variation), indicating that the effect of diet varied over the rearing period. Conversely, larvae reared on whole oat flakes showed lower growth than those on OB or WB, indicating that the base substrate strongly influences performance. The addition of OWW further reduced mean larval weight (Table 1) particularly in the OF+ group, emphasizing the combined role of substrate and supplementation. This effect became statistically significant across all diet groups by day 30. The decline in growth performance after 45 days was particularly notable, ranging from 20.1% for WB+ to 40.3% and 44.2% for OB+ and OF+, respectively. In all OWW-enriched diets (OB+, WB+, OF+), the observed increase in antioxidant compounds and activity reflects the high phenolic content of OWW, a waste stream known to contain elevated levels of hydroxytyrosol, tyrosol, and other phenolics with strong radical scavenging potential. However, the same bioactive compounds that confer antioxidant capacity can also exhibit phytotoxic or antinutritional effects in vivo, potentially explaining the negative impact on larval growth at the tested inclusion level [40].

**Figure 2:**
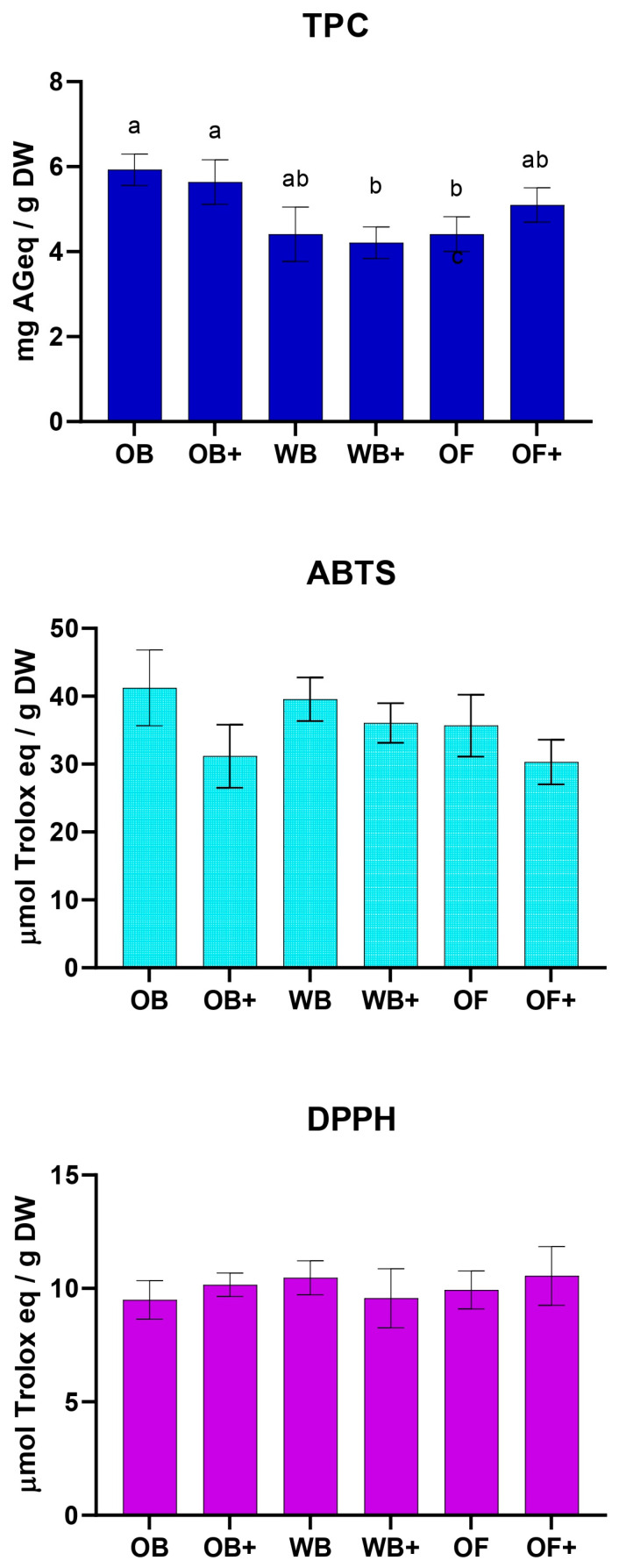
Total phenolic content (TPC) and antioxidant activity measured by ABTS and DPPH assays in *Tenebrio molitor* larvae fed oat bran (OB), wheat bran (WB), whole oat flakes (OF), or OWW-enriched diets (OB+, WB+, OF+) after 45 days of rearing. TPC was expressed as milligrams of gallic acid equivalents per gram of dry weight (mg GAE/g DW), antioxidant capacity was expressed as µmol of Trolox equivalents per gram of dry weight (µmol TE/g DW). Results are expressed as mean ± SD of at least three independent experiments. Statistical analysis was performed using one-way ANOVA followed by Tukey’s post hoc test. Values labeled with different letters (a, b) are significantly different (*p* < 0.05).

Despite the important antioxidant activity of the enriched diets, after 45 days of rearing, no statistically significant differences were observed between the TPC of TML fed the standard diets and those fed the OWW-enriched diets (Figure 2). Similar results were obtained for TAC measured using both ABTS and DPPH assays.

**Figure 3:**
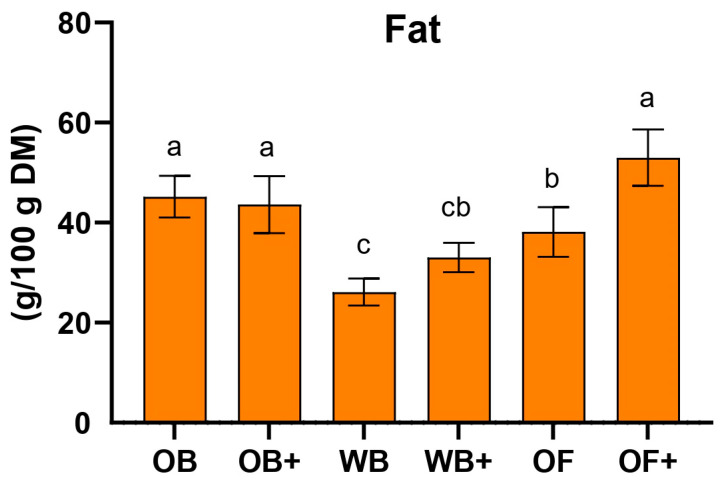
Quantification of fat content (g fat/100 g dry matter) in *Tenebrio molitor* larvae fed wheat bran (WB), oat bran (OB), oat flakes (OF), or diets enriched with olive mill wastewater (WB+, OB+, OF+) after 45 days of rearing. Values represent the mean ± SD of at least three independent experiments. Statistical analysis was performed using one-way ANOVA followed by Tukey’s post hoc test. Values with different letters (a, b, c) are significantly different (*p* < 0.05).

Body fat content is an important physiological trait associated with TML growth, and previous studies have shown that increased fat accumulation often correlates with reduced growth performance [41]. In the present study, fat quantification revealed a slight increase in the WB+ group and a statistically significant increase in the OF+ group, which also showed lower growth performance compared to their respective controls (Figure 3). Similar patterns have been reported in other studies where TML were reared on agro-industrial by-products. For example, diets based on alternative substrates such as olive pomace or other plant-derived residues have been shown to alter larval body composition, often leading to increased lipid accumulation when growth performance is reduced [42]. Likewise, studies evaluating the use of various agricultural by-products in mealworm diets have reported significant variations in total lipid content depending on substrate composition, indicating that nutrient imbalance or reduced dietary quality may promote lipid storage rather than biomass growth [43]. These findings suggest that the higher fat levels observed in the OF+ group may reflect metabolic adjustments to diet composition rather than improved nutritional status, as lipid accumulation can occur when larvae experience suboptimal growth conditions.

The fatty acid profile of larval fat was also analyzed to assess lipid quality. As shown in Table 2, all larvae fed with OWW-enriched diets exhibited a general decrease in polyunsaturated fatty acids (PUFA) in favor of monounsaturated fatty acids (MUFA). Despite this shift, lipid quality indices including the atherogenic index (AI), thrombogenic index (TI), hypocholesterolemic/hypercholesterolemic ratio (HH), and health-promoting index (HPI) remained comparable to those of larvae reared on the standard diets. Similar diet-dependent changes in polyphenol content, fat levels, and fatty acid profiles have been reported in TML, demonstrating that the composition of dietary substrates strongly influences both the accumulation of bioactive compounds and the relative proportions of lipid classes. For example, supplementation with nursery wastes leaves significantly increased total polyphenols (up to 5–7 times higher than standard diets) maintained total fat content, and improved fatty acid profiles by increasing PUFA lowering the n6/n3 ratio. Likewise, distillery by-products, citrus residues, and algae-enriched diets increased polyphenol content and antioxidant activity, as well as modifying MUFA and PUFA proportions, reflecting the larvae’s ability to adjust lipid metabolism according to the nutritional composition of the rearing substrate [19,20,44]. These findings suggest that variations in PUFA and other fatty acids observed in supplemented diets are likely diet-induced metabolic adjustments rather than a reduction in lipid nutritional quality, as overall health-related lipid indices remained stable or improved.

**Figure 4:**
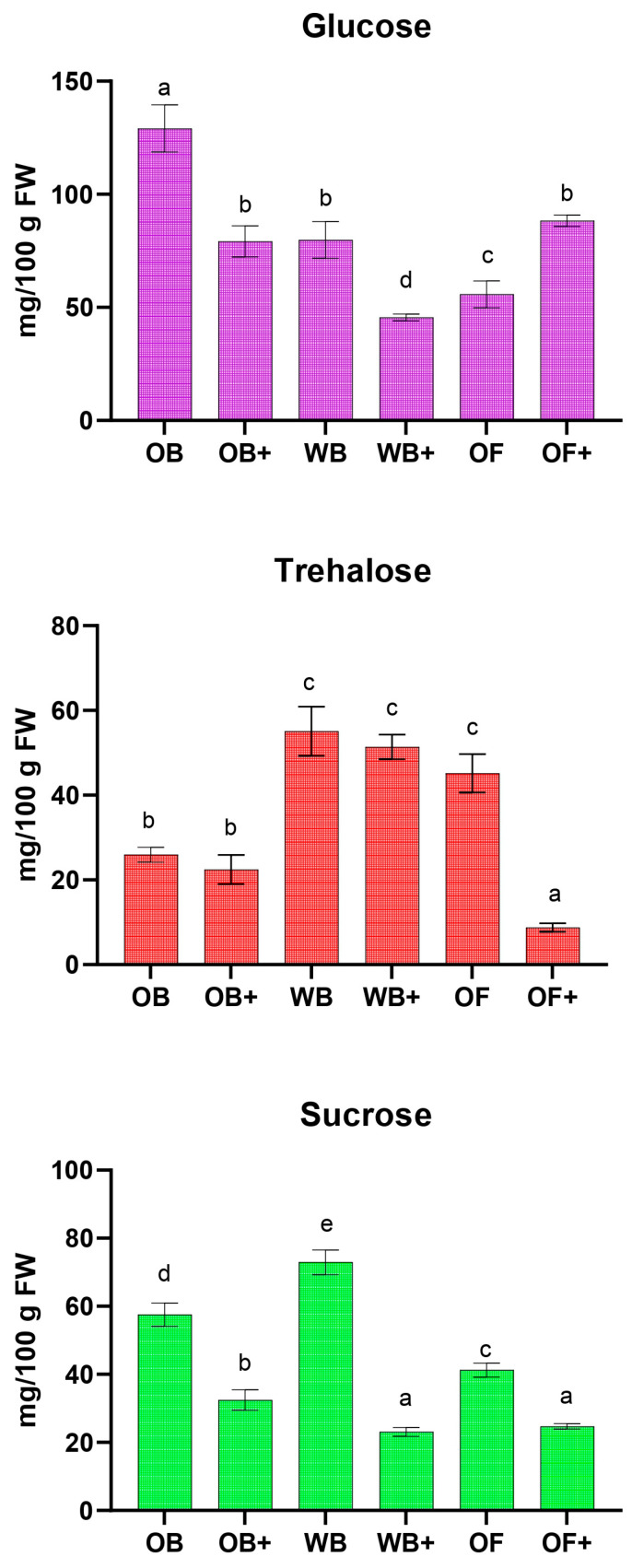
Quantification of the main carbohydrates (mg/100 g dry weight) in *Tenebrio molitor* larvae fed with oat bran (OB), wheat bran (WB), whole oat flakes (OF), or diets supplemented with olive mill wastewater (OB+, WB+, OF+) after 45 days of rearing. Data represent the mean ± SD of at least three independent experiments. Statistical analysis was performed using one-way ANOVA followed by Tukey’s post hoc test. Different letters (a, b, c, d, e) indicate significant differences within the same time point (*p* < 0.05).

A targeted metabolomic analysis was performed using NMR spectroscopy to investigate the biochemical impact of diets enriched with olive mill wastewaters (OWWs) on TML. This approach allowed the quantification of key metabolites involved in energy metabolism and nutrient utilization, including carbohydrates (glucose, trehalose, sucrose), essential amino acids, and organic acids (Krebs cycle intermediates and lactate) after 45 days of rearing. Even among standard diets (OB, WB, OF), subtle differences were observed in glucose, trehalose, sucrose, and essential amino acids such as leucine, isoleucine, and valine, indicating that substrate composition alone can influence energy metabolism and nutrient allocation. As shown in Figure 4, OWW supplementation amplified these effects: trehalose and sucrose consistently decreased in larvae fed OWW-enriched oat bran (OB+) and wheat bran (WB+), while in OF+, glucose increased and trehalose decreased, correlating with lower growth performance and suggesting enhanced energy utilization or stress-induced metabolic demand. These metabolic changes complement the observed diet-dependent shifts in polyphenol accumulation, fat content, and fatty acid composition, highlighting that diet composition can simultaneously modulate bioactive compound uptake, lipid metabolism, and energy pathways in TML.

**Figure 5:**
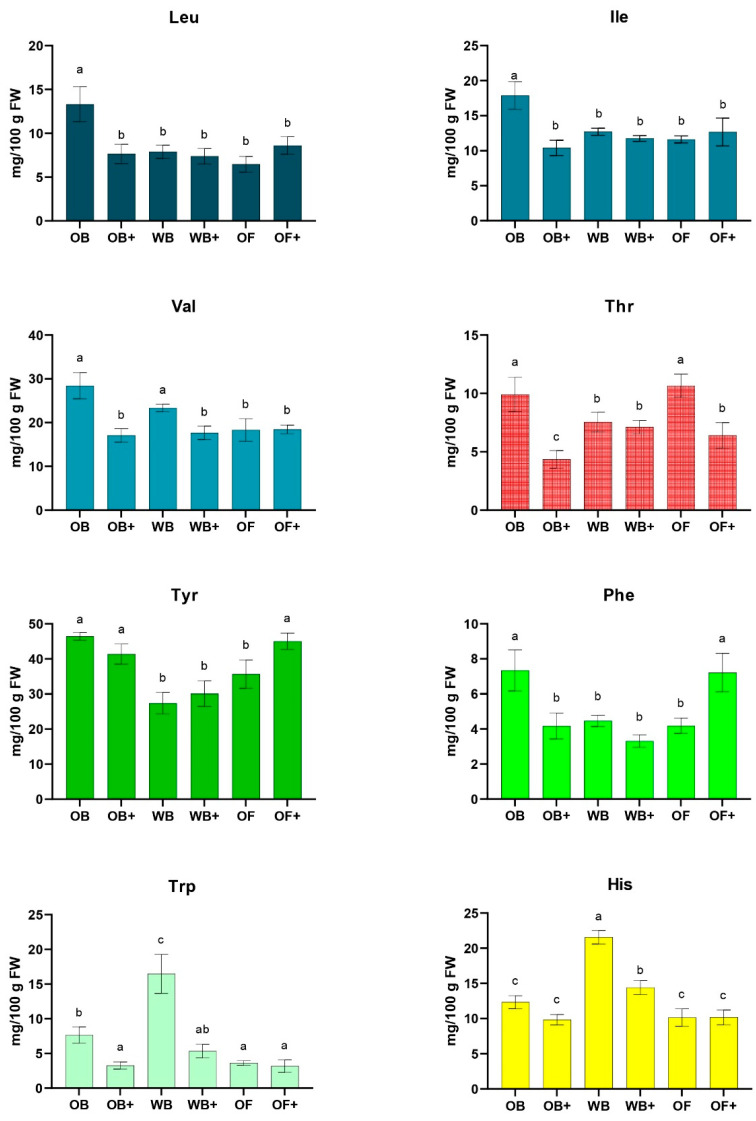
Quantification of the main essential amino acids (mg/100 g dry weight) in *Tenebrio molitor* larvae fed with oat bran (OB), wheat bran (WB), whole oat flakes (OF), or diets supplemented with olive mill wastewater (OB+, WB+, OF+) after 45 days of rearing. Data represent the mean ± SD of at least three independent experiments. Statistical analysis was performed using one-way ANOVA followed by Tukey’s post hoc test. Different letters (a, b, c) indicate significant differences within the same time point (*p* < 0.05).

Essential amino acids (Figure 5), particularly branched-chain amino acids (Leu, Ile, Val), showed significant reductions in OB+ and, to a lesser extent, in WB+. Aromatic amino acids (Tyr, Phe, Trp) were also reduced in OB+, indicating that phenolic-rich OWW may modulate amino acid availability or protein turnover, possibly reallocating resources toward detoxification or antioxidant defense pathways. Tryptophan and tyrosine are not only involved in protein synthesis but also serve as precursors for catecholamines required for cuticle sclerotization and melanization, processes critical for proper molting and exoskeleton formation in insects [45,46]. Threonine, significantly decreased in OB+ and OF+, is a major component of chitin-binding proteins and chitinases involved in cuticle remodeling, indicating a potential impact on molting and larval growth [47]. Histidine levels were largely maintained except in WB+, consistent with its role in oxidative stress protection and protein stabilization [45]. These results suggest that the addition of OWW can selectively influence amino acid composition, potentially affecting both growth performance and cuticle development in *Tenebrio molitor,* as also supported by studies demonstrating the importance of dietary amino acids for larval growth and development [48,49,50].

**Figure 6:**
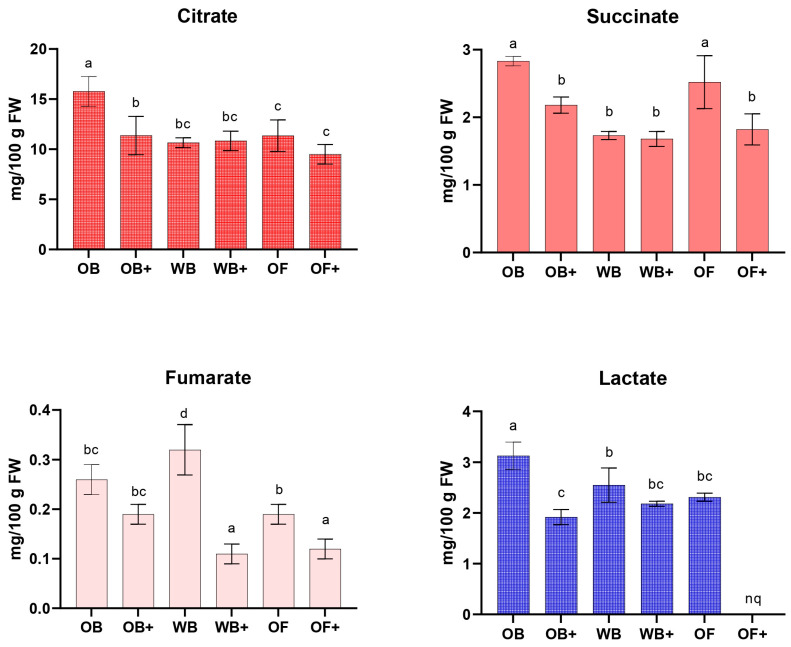
Quantification of the main organic acids (mg/100 g dry weight) in *Tenebrio molitor* larvae fed with oat bran (OB), wheat bran (WB), whole oat flakes (OF), or diets supplemented with olive mill wastewater (OB+, WB+, OF+) after 45 days of rearing. nq: not quantified, data below the limit of quantification. Data represent the mean ± SD of at least three independent experiments. Statistical analysis was performed using one-way ANOVA followed by Tukey’s post hoc test. Different letters (a, b, c, d) indicate significant differences within the same time point (*p* < 0.05).

As reported in Figure 6, organic acids involved in central carbon metabolism, including citrate, succinate, and fumarate, were generally decreased in larvae fed enriched diets, and lactate was notably low in OF+, reflecting altered energy metabolism and potential shifts toward lipid storage rather than growth [51,52].

These metabolomic patterns align with growth and compositional data. In fact, larvae on OB+ and OF+ diets exhibited reduced weight gain and increased lipid accumulation, while WB+ showed milder effects. This indicates that metabolic adjustments are closely associated with performance outcomes, highlighting that bioactive enrichment must be considered alongside optimal rearing conditions to ensure adequate larval growth and industrially relevant productivity. Comparable NMR-based studies on TML and other insects indicate that bioactive dietary components, such as phenolic-rich plant byproducts, can influence carbohydrate and amino acid metabolism, modify central energy pathways, and impact growth and antioxidant capacity [51]. Thus, this integrated metabolomic assessment highlights how the inclusion of OWW alters nutrient allocation and metabolic state in mealworms, providing mechanistic insight into the observed changes in growth, lipid accumulation, and stress-related metabolites. The observed reduction in larval growth upon supplementation with OWW is consistent with previous studies on TML fed with olive pomace or other olive by-products. For example, Ruschioni et al. [42] reported that moderate inclusion of olive pomace in TML diets could maintain growth performance close to control diets, whereas higher proportions led to a significant decrease in larval weight and prolonged development time. These effects have been attributed to the high content of fiber and bioactive compounds, particularly phenolics, which may reduce nutrient digestibility and absorption, ultimately impairing growth despite adequate caloric intake. Similarly, in our trials, larvae fed OWW-enriched diets displayed lower weight gain, particularly in the oat-based substrates, while lipid accumulation increased, suggesting a possible shift in nutrient partitioning toward storage rather than somatic growth. These findings align with previous observations that olive-derived by-products can provide bioactive molecules but may also impose metabolic constraints, highlighting the need to balance enrichment with larval performance when valorizing agro-industrial residues in insect rearing [42,52]. This limitation is not unique to olive by-products. Previous studies have shown that TML growth and rearing efficiency can be negatively affected by diets based on various agro-industrial by-products, such as oilseed press cakes, grape seeds, and other industrial residues [53,54,55,56]. These substrates often differ in nutrient composition, fiber content, and antinutritional factors, which can compromise larval weight gain, feed conversion efficiency, and fatty acid profiles. The effects are particularly pronounced when a single by-product constitutes the majority or entirety of the diet, as observed in trials with milk industry by-products or *Chaetomorpha linum* at 100% inclusion, where larvae exhibited reduced growth and poorer performance compared to more balanced, cereal-based diets [24,25]. Collectively, these findings indicate that while by-products can be valorized as functional feed components, careful formulation is essential to maintain larval productivity and nutritional quality.

Overall, these findings confirm that OWW can be valorized as a functional dietary supplement, increasing the content of bioactive compounds in the feed, but the inclusion must be carefully optimized to avoid compromising larval growth. Similar observations have been reported in other insect models, where high-fiber or high-phenolic agro-industrial by-products increased stress-related metabolites and altered energy allocation, reducing growth performance while enhancing certain biochemical traits [19,51,57]. This highlights the potential of metabolomic profiling to elucidate the physiological and biochemical impacts of alternative substrates in insect rearing.

The presence of phenolic compounds in OWW represents a critical factor in understanding their effects when used as feed supplements for TML. OWW are rich in a diverse range of phenolics, including hydroxytyrosol, tyrosol, and various phenolic acids and derivatives, often reaching concentrations up to tens of grams per liter [42,57]. These compounds are highly water-soluble, contributing to the well-documented phytotoxic and antimicrobial properties of OWW [42,57,58].

Phenolics in OWW have been shown to inhibit seed germination, root elongation, and vegetative growth in various plant bioassays, with complex mixtures of phenolics generally exhibiting stronger inhibitory effects than individual purified compounds [2,42]. This suggests that synergistic interactions among multiple phenolic molecules and other metabolites enhance the overall toxicity of the wastewater. Moreover, hydroxytyrosol and tyrosol exhibit antibacterial and antifungal activity, which can interfere with microbial populations in the gut that might otherwise contribute to substrate nutrient availability or digestion processes [59,60,61].

In the context of insect rearing, the high phenolic content of OWW may act as an antinutritional factor. Although few studies have directly evaluated olive mill by-products as feed for insects, the reduced growth performance observed in TML fed diets supplemented with OWW can reasonably be associated with the inhibitory effects of these phenolic compounds. The decrease in larval weight and alterations in metabolite profiles (including carbohydrates, amino acids, and organic acids) may reflect the combined effects of reduced nutrient digestibility, mild oxidative stress, or interference with microbial communities involved in digestion. Similar inhibitory effects of high phenolic diets have been documented in other organisms, including fish and invertebrates, where excessive phenolics limit growth and feed conversion efficiency [62,63].

Therefore, while OWW supplementation enhances the antioxidant content of the diet, it also introduces bioactive phenolic compounds that likely act as antinutrients, moderating larval growth and metabolic performance. This dual effect highlights the need to balance the potential nutritional and bioactive benefits of OWW against its inhibitory properties when formulating insect diets.

#### Multivariate Analysis

As reported in Figure S1, unsupervised Principal Component Analysis (PCA) was initially performed including all six experimental groups (OB, OB+, WB, WB+, OF, OF+). The first two principal components explained approximately 50% of the total variance (PC1 of 29%, PC2 of 21%). The score plot revealed a clear clustering pattern primarily driven by basal diet, with samples segregating according to OB, WB, and OF dietary regimens. Within each dietary cluster, STD (OB, WB, OF) and OWW enriched samples (OB+, WB+, OF+) were positioned in close proximity, although a subtle displacement was observable. The multivariate analysis revealed a hierarchical structure of metabolic determinants. PCA including all experimental groups demonstrated that basal diet was the principal driver of global variance. This finding is consistent with the well-established influence of macronutrient composition and dietary matrix on systemic metabolic organization. The separation observed along PC1 suggests that differences in substrate availability and metabolic routing associated with OB, WB, and OF diets shape the primary axes of metabolic variability. Such diet-driven clustering is expected in datasets where nutritional composition modulates carbohydrate flux, amino acid turnover, and lipid remodeling.

**Figure 7:**
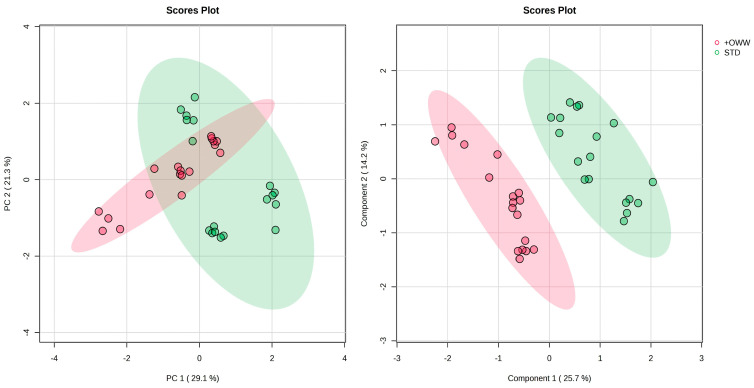
Score plots of Principal Component Analysis (PCA, **left**) and Partial Least Squares Discriminant Analysis (PLS-DA, **right**). STD diets (OB, WB, OF), +OWW diets (OB+, WB+, OF+).

To better evaluate the treatment effect independently of diet-driven variability, samples were regrouped according to supplementation STD (OB, WB, OF) vs. OWW enriched samples +OWW (OB+, WB+, OF+) and re-analyzed by PCA (Figure 7). In this configuration, a partial but consistent separation along PC1 was observed between STD and +OWW groups. Although confidence ellipses exhibited partial overlap, centroid displacement suggested a systematic treatment-associated shift in metabolic space. Although the effect size was moderate in the unsupervised model, the directional consistency across dietary backgrounds indicates that OWW supplementation induces a transversal metabolic modulation. This pattern suggests that OWW does not override basal dietary programming but superimposes a secondary metabolic adjustment across distinct nutritional contexts.

To further assess treatment-related metabolic modulation, Partial Least Squares–Discriminant Analysis (PLS-DA) was performed comparing STD vs. OWW samples (*n* = 18 per group). The model showed strong classification performance with R^2^ of 0.97, Q^2^ of 0.89 and cross-validation accuracy close to 1.0 (Figure S2). Permutation testing (1000 permutations) confirmed model robustness (*p* = 0.001), indicating that class separation was not due to random overfitting. The robust performance of the PLS-DA model confirms that treatment-related metabolic differences are structured and reproducible (Figure 7). The high Q^2^ value indicates strong predictive capability, while permutation testing excludes model overfitting.

The supervised model therefore demonstrates that OWW supplementation exerts a statistically significant and biologically consistent metabolic shift across all diets. The discrepancy between moderate separation in PCA and strong discrimination in PLS-DA is methodologically coherent. PCA maximizes total variance, predominantly reflecting diet-driven variability, whereas PLS-DA optimizes covariance between metabolite abundance and treatment class, thereby isolating treatment-specific effects.

**Figure 8:**
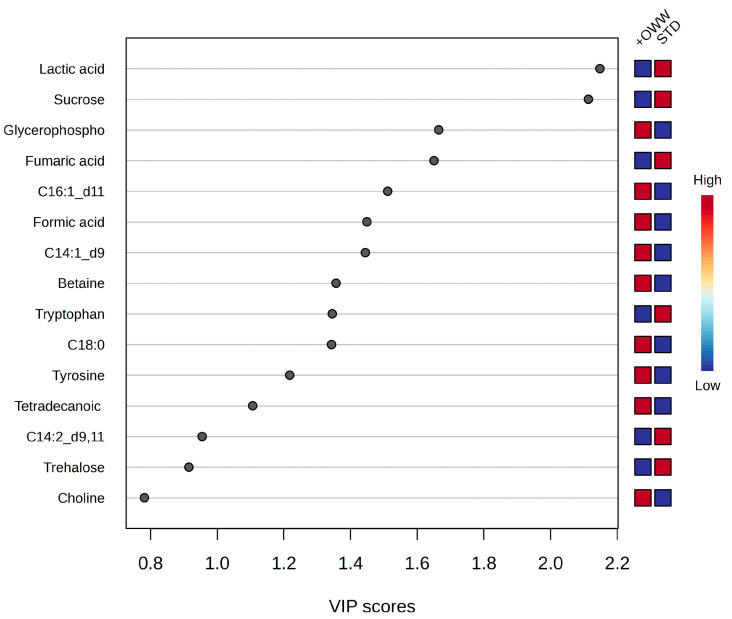
Analysis of the Variable Importance in Projection (VIP) scores derived from the PLS-DA model for larvae analyzed under different diets.

As reported in Figure 8, Variable Importance in Projection (VIP) analysis identified several metabolites contributing significantly to group discrimination (VIP > 1), including sucrose, glycerophosphocholine, fumaric acid, tryptophan, betaine, and selected fatty acid derivatives. VIP-ranked metabolites suggest that OWW supplementation influences multiple metabolic axes, in particular, alterations in fumaric acid and sucrose indicate modulation of central carbon metabolism, potentially reflecting shifts in glycolytic flux and mitochondrial activity. Changes in TCA intermediates may denote altered oxidative metabolism or substrate utilization efficiency, while Tryptophan modulation may reflect alterations in nitrogen balance, gut-host interactions, or signaling pathways linked to immune or oxidative responses. Given the central role of tryptophan as a precursor for serotonin and as a substrate for the kynurenine pathway, alterations in its levels may have broader regulatory implications, including effects on neurophysiological functions, stress responses, and immune modulation in insects. Similar roles for tryptophan metabolism have been described in insect models, where serotonin influences feeding behavior and stress responses [64] and kynurenine pathway intermediates modulate immune and oxidative responses [65,66].

Changes in glycerophosphocholine and betaine suggest modifications in phospholipid remodeling and one-carbon metabolism. Betaine is a key methyl donor and osmolyte, indicating possible epigenetic or osmoprotective adjustments.

Collectively, these alterations suggest that OWW supplementation induces a coordinated metabolic adaptation involving energy homeostasis, redox balance, and membrane-related processes.

The integrated multivariate results support a model in which basal diet establishes the primary metabolic framework, while OWW supplementation introduces a secondary, yet statistically robust, modulation layer. Importantly, the treatment effect appears consistent across dietary backgrounds, as evidenced by the successful binary classification (STD vs. OWW) despite underlying diet heterogeneity. This indicates that OWW acts as a transversal metabolic modulator rather than a diet-specific perturbator. Such a pattern is consistent with functional ingredients or bioactive compounds that fine-tune metabolic networks rather than inducing wholesale metabolic reprogramming.

## 3. Materials and Methods

The solvents and reagents used (analytical grade) were obtained from Sigma-Aldrich S.r.l. (Milan, Italy) and used without additional purification. Milli-Q quality water (Millipore, Milford, MA, USA) was used. The olive mill wastewaters (OWWs) used in this study were collected during the 2024 olive campaign in the province of Siena (Italy) from the following mills: Moliture Ascianesi S.r.l. (via Martiri della Libertà, Asciano, SI), Frantoio La Romita (Via Umberto Primo, Montisi, SI), and Frantoio Mazzarrini (Rigomagno Scalo, Sinalunga, SI). For the rearing trials, each treatment was set up with three replicates, each consisting of 50 TML (approximately 12 mm in length and weighing 10–20 mg), placed in aluminum trays containing 10 g of substrate. Twice a week, a slice of carrot (2 g) was added to provide water for the larvae. The trays were randomly assigned to treatments in a completely randomized design. Larvae were reared under semi-dark conditions in a climate-controlled room at 27 ± 1 °C and 40–50% relative humidity. The trials were conducted over 15, 30, and 45 days, with larval survival and body weight monitored at each time point. Prior to freezing at −18 °C for storage, the larvae were washed with deionized water and subjected to a 24 h fasting period. Larval weight was determined by collectively weighing all larvae within each tray (50 larvae per tray). Each diet was tested in triplicate trays, and the mean larval weight per tray was calculated by dividing total tray weight by the number of larvae. Reported values represent the average ± SD of the three replicates.

### 3.1. Formulation of Diets

For the feeding trials, standard diets (STD) were selected as references—wheat bran (WB, Organic Sarchio, Carpi, Italy), oat bran (OB, Cèreal, Milan, Italy), and whole oat flakes (OF, Stella Foods s.r.l., Rivalta di Torino, Italy)—chosen for their similar composition and suitability for TML rearing (Table S3). 180 g of commercial substrate of each reference diet was enriched with 500 mL of olive mill wastewater (OWW) prepared by mixing equal parts of the three OWW types analyzed (OWW1–OWW3). The OWW supplementation accounted for 20% of the total mixture on a dry weight basis. The corresponding fortified diets (+OWW: WB+, OB+, OF+) were dried under a nitrogen stream at 40 °C to ensure proper storage and use (residual moisture below 15%). Prior to feeding, the diets were ground and homogenized using a domestic blender TSM6A013B (Bosh, Gerlingen-Schillerhoehe, Germany) and sieved through a 500 µm Endecotts test sieve (Endecotts Ltd., London, UK).

### 3.2. Determination of Physicochemical Parameters of OWW

The pH of olive mill wastewater (OWW) samples (OWW1–OWW3) was measured using an Amel Model 234 Titrator equipped with a combined electrode. Total solids (TS) were determined gravimetrically by placing a precisely weighed 5 mL aliquot of each sample in a pre-weighed porcelain dish, followed by drying in an oven at 105 ± 2 °C for 48 h. TS values were calculated based on the weight difference and expressed as g/L of sample. Total suspended solids (TSS) were assessed by filtering 5 mL of each sample through 0.45 µm cellulose acetate filters (Millipore). Filters were pre-weighed, dried at 105 °C for 24 h, and re-weighed to calculate TSS, expressed as g/L.

The lipophilic fraction of each sample was extracted by acidifying 20 mL aliquots with 1 N HCl to pH 2, followed by three successive extractions with 20 mL n-hexane. The organic fractions were combined, and the solvent was removed under reduced pressure. The resulting lipid extract was weighed to determine the residual fat content and stored at −18 °C until further analyses. The hydrophilic fraction was obtained from the defatted wastewater by performing three successive extractions with 20 mL ethyl acetate. The combined organic layers were evaporated under reduced pressure, and the extracts were stored at −18 °C until subsequent analyses. All the determinations were performed in triplicate, using larvae from three separate trays for each treatment.

### 3.3. Proximate Composition

Moisture content was determined by drying 1.0 g of TML in a ventilated oven at 105 °C for 14–16 h until constant weight was achieved. Water content was calculated as the difference between the initial and final sample weights and expressed as percentage of fresh weight [67].

Total nitrogen was quantified on 1.0 g of dried sample of OWW using the Kjeldahl method. Crude protein content was calculated by applying a nitrogen-to-protein conversion factor of 6.25 for OWW samples.

Total lipid content was determined according to the Folch method. Briefly, 5 g of sample (OWW or TML) were homogenized for 5 min using an IKA Labortechnik T25 basic homogenizer (IKA WERKE GmbH & Co., Staufen, Germany) and extracted with chloroform (CHCl_3_). The organic phase was filtered and washed twice with the upper phase consisting of CHCl_3_/CH_3_OH/H_2_O (53:27:20, *v*/*v*/*v*). The extract was subsequently washed with 10 mL of 0.7% KCl solution to remove non-lipid contaminants. After solvent evaporation, total fat content was determined gravimetrically prior to fatty acid (FA) analysis. All the analyses were performed in triplicate, using OWW or larvae from three separate trays for each treatment.

### 3.4. Preparation of Extracts for Metabolomic Analysis

Lipid and hydrophilic extractions from TML were obtained according to the Bligh–Dyer method with minor modifications. Briefly, 1.0 g of frozen TML sample was mixed with 2 mL of CHCl_3_ and 2 mL of MeOH and homogenized for 2 min using an IKA Labortechnik T25 basic homogenizer (IKA WERKE GmbH & Co.). Finally, 1 mL of distilled water was added, and the mixture was homogenized for a further 30 s to promote phase separation. The resulting mixture was filtered and the two phases collected. The solvent was evaporated under a nitrogen stream and stored at −18 °C prior analysis.

### 3.5. Quantification of Antioxidant Compounds and Antioxidant Activity

Total polyphenol content (TPC) was determined using the Folin–Ciocalteu colorimetric method, with gallic acid employed as the calibration standard, as previously described [25]. Results were expressed as milligrams of gallic acid equivalents per gram of dry weight (mg GAE/g DW).

Radical scavenging activity was evaluated using the 1,1-diphenyl-2-picrylhydrazyl (DPPH) assay according to the procedure reported in [18], with Trolox used as the reference antioxidant. Antioxidant capacity was expressed as Trolox equivalent antioxidant capacity (TEAC), reported in µmol of Trolox equivalents per gram of dry weight (µmol TE/g DW).

### 3.6. ^1^H NMR Analysis

For the analysis of lipophilic extracts, 20 mg of sample were dissolved in 0.7 mL of CDCl_3_ containing 0.03% (*v*/*v*) tetramethylsilane (TMS; Sigma-Aldrich, Darmstadt, Germany) as internal standard. One-dimensional ^1^H NMR spectra were acquired at 298 K using a Bruker Avance DPX 400 MHz spectrometer (Bruker Biospin, Rheinstetten, Germany). Spectra were recorded using a standard single-pulse sequence with a 90° flip angle, four dummy scans, a relaxation delay of 3 s, and 16 acquisition scans.

Samples were dissolved in 1.0 mL of 400 mM phosphate buffer prepared in D_2_O (pH 7.0), containing 1 mM 3-(trimethylsilyl)propionic acid sodium salt (TSP) as a chemical shift reference. Prior to analysis, samples were filtered and analyzed using the Bruker zgpr pulse sequence.

Signal assignment in the ^1^H NMR spectra was carried out by comparison with literature data [24,51,52] and by using the Chenomx NMR Suite (v11, Chenomx Inc., Edmonton, AB, Canada). Assignments are reported in Table S4. For NMR analyses, measurements were conducted in sextuplicate by subdividing larvae from the same trays.

### 3.7. Quantification of Fatty Acids

Fatty acids (10 mg of lipid extract) were converted to their corresponding methyl esters (FAMEs) by transesterification with 1 mL of boron trifluoride (BF_3_) in methanol, incubated at 100 °C for 1 h, as previously described [67].

Gas chromatography analysis was performed using a PerkinElmer Clarus 500 GC system (PerkinElmer, Norwalk, CT, USA) equipped with a flame ionization detector (GC-FID) and a split/splitless injector, following the method reported in [67]. Fatty acids were identified by comparison with reference standards and quantified based on the relative peak areas of the resolved FAMEs. Results were expressed as percentage of total detected fatty acids.

### 3.8. Determination of Lipid Quality Indices

Lipid quality indices associated with cardiovascular risk, including the index of thrombogenicity (TI), index of atherogenicity (AI), hypocholesterolemic/hypercholesterolemic ratio (H/H), and health-promoting index (HPI), were calculated according to the equations reported in the literature [68,69,70].

$$TI=\frac{\left(C14:0)+(C16:0)+(C18:0\right)}{\left(0.5\times MUFA)+(0.5\times \omega 6)+(3\times \omega 3)+(\omega 3/\omega 6\right)}$$

$$AI=\frac{\left(C12:0)+(4\times C14:0)+(C16:0\right)}{PUFA(\omega 6+\omega 3)+MUFA}$$

$$HH=\frac{cis\text{-}C18:1+\Sigma PUFA}{C12:0+C14:0+C16:0}$$

$$HPI=\frac{\Sigma PUFA}{C12:0+(4\times C14:0)+C16:0}$$

### 3.9. Statistical Analysis

All experimental data were analyzed using GraphPad Prism 8.2 (GraphPad Software, La Jolla, CA, USA) and are reported as mean ± standard deviation (SD). Homogeneity of variances was assessed by the Brown-Forsythe test, and normality of data distribution was verified using the Shapiro–Wilk test. Larval body weight has been analyzed with two-way ANOVA with different diets and time of rearing as fixed factors. For the analysis of nutrients, lipids, fatty acids and metabolites, including organic acids, sugars and amino acids, one-way ANOVA was performed, followed by Tukey’s post hoc test for multiple comparisons. Differences were considered statistically significant at *p* < 0.05.

## 4. Conclusions

Olive mill wastewater (OWW) is a by-product rich in phenolic compounds and residual lipids, able to increase the antioxidant content of feeds but not enhance antioxidant activity in *Tenebrio molitor* larvae (TML). At the tested inclusion level, OWW supplementation reduced larval growth and altered nutrient allocation, resulting in modest increases in lipid accumulation. Metabolomic analyses indicated shifts in carbohydrate, amino acid, and organic acid profiles, reflecting adjustments in energy metabolism and biochemical pathways. These results highlight that, although OWW acts as a bioactive dietary modulator, its use at this level is not suitable for promoting larval growth in industrial TML rearing. Nevertheless, the observed metabolic effects may be of interest for future functional studies, including the valorization of OWW in the food or nutraceutical contexts.

## Figures and Tables

**Table 1:** Average weight of *Tenebrio molitor* larvae at different rearing days on oat bran (OB), wheat bran (WB), oat flakes (OF), or diets supplemented with olive mill wastewater (OB+, WB+, OF+).

Diet	Days			
	0	15	30	45
	Mean ± SD	Mean ± SD	Mean ± SD	Mean ± SD
OB	12.27 ± 1.01 ^a^	19.23 ± 2.12 ^a^	33.06 ± 2.67 ^c^	72.97 ± 0.69 ^f^
OB+	11.31 ± 2.17 ^a^	18.03 ± 3.14 ^b^	25.17 ± 2.95 ^b^	43.56 ± 7.03 ^d^
WB	12.62 ± 3.92 ^a^	33.29 ± 2.56 ^c^	45.94 ± 1.89 ^d^	99.44 ± 7.29 ^g^
WB+	11.48 ± 1.50 ^a^	20.10 ± 1.77 ^b^	32.54 ± 3.14 ^c^	79.42 ± 8.37 ^f^
OF	12.84 ± 2.94 ^a^	20.14 ± 2.09 ^b^	35.09 ± 2.74 ^c^	63.26 ± 6.71 ^e^
OF+	10.77 ± 1.82 ^a^	17.80 ± 1.97 ^b^	27.18 ± 1.88 ^b^	35.32 ± 5.70 ^c^

Results represent the mean ± SD of three experiments. Statistical analysis was performed with two-way ANOVA with different diets and time of rearing (0, 15, 30, and 45 days) as fixed factors and Tukey’s post hoc test. a, b, c, d, e, f and g values without a common superscript are significantly different (*p* < 0.05).

**Table 2:** Fatty acid composition (%) after 45 days of rearing on oat bran (OB), wheat bran (WB), oat flakes (OF), or diets supplemented with olive mill wastewater (OB+, WB+, OF+). Results represent the mean ± SD of three experiments. Values with different letters (a, b, c) within the same row are significantly different (*p* < 0.05). nd: Not determined. SFA: short chain fatty acids; MUFA: monounsaturated fatty acids; PUFA: polyunsaturated fatty acids. AI: Atherogenic Index; TI: Thrombogenic Index; HH: Hypocholesterolemic/Hypercholesterolemic ratio; HPI: Health-Promoting Index.

Fatty Acid	OB	OB+	WB	WB+	OF	OF+
	Mean ± SD	Mean ± SD	Mean ± SD	Mean ± SD	Mean ± SD	Mean ± SD
C12:0	0.253 ± 0.099	0.255 ± 0.097	0.308 ± 0.063	0.375 ± 0.072	0.251 ± 0.034	0.325 ± 0.086
C13:0	nd	nd	0.031 ± 0.010 ^b^	0.079 ± 0.015 ^c^	0.007 ± 0.012 ^a^	0.039 ± 0.009 ^b^
C14:0	3.806 ± 0.486	3.961 ± 0.527	3.775 ± 0.581	4.307 ± 0.816	3.923 ± 0.308	4.273 ± 0.538
C15:0	0.031 ± 0.000 ^b^	0.017 ± 0.012 ^a^	0.024 ± 0.006 ^a^	0.048 ± 0.006 ^b^	0.017 ± 0.005 ^a^	0.036 ± 0.006 ^b^
C16:0	13.619 ± 1.536 ^b^	13.617 ± 1.253 ^b^	10.982 ± 0.771 ^a^	13.597 ± 0.149 ^b^	12.655 ± 0.728 ^ab^	11.568 ± 0.644 ^a^
C17:0	0.017 ± 0.029	0.120 ± 0.101	0.051 ± 0.018	0.075 ± 0.078	0.040 ± 0.020	0.070 ± 0.028
C18:0	3.676 ± 0.136	2.779 ± 1.954	3.443 ± 0.397	3.095 ± 0.163	3.595 ± 0.049	3.168 ± 0.030
C20:0	0.126 ± 0.048	0.159 ± 0.013	0.116 ± 0.021	0.114 ± 0.017	0.131 ± 0.057	0.112 ± 0.015
Σ SFA	21.529 ± 2.073	20.907 ± 2.113	18.730 ± 0.341	21.691 ± 0.984	20.619 ± 0.928	19.866 ± 0.973
C14:1 Δ11	0.355 ± 0.040	0.332 ± 0.146	0.376 ± 0.088	0.231 ± 0.067	0.449 ± 0.025	0.401 ± 0.094
C14:1 Δ9	0.031 ± 0.000 ^a^	0.034 ± 0.006 ^a^	0.062 ± 0.000 ^b^	0.134 ± 0.010 ^c^	0.067 ± 0.006 ^b^	0.046 ± 0.011 ^ab^
C16:1 Δ9	1.716 ± 0.230 ^a^	1.700 ± 0.231 ^a^	1.638 ± 0.019 ^a^	2.449 ± 0.158 ^b^	1.620 ± 0.162 ^a^	2.300 ± 0.206 ^b^
C16:1 Δ11	1.104 ± 0.286	1.170 ± 0.313	1.364 ± 0.143	0.892 ± 0.158	1.351 ± 0.075	0.863 ± 0.090
C18:1 Δ9	51.391 ± 2.547	50.966 ± 4.522	51.133 ± 2.510	41.856 ± 0.853	50.101 ± 1.921	48.110 ± 3.322
C20:1 Δ11	0.072 ± 0.000	0.024 ± 0.012	0.065 ± 0.006	0.086 ± 0.025	0.064 ± 0.086	nd
Σ MUFA	52.942 ± 1.523 ^b^	51.996 ± 2.698 ^b^	55.240 ± 1.321 ^c^	46.120 ± 0.574 ^a^	53.950 ± 1.186 ^b^	51.720 ± 3.254 ^b^
C14:2 Δ9,11	0.065 ± 0.016 ^a^	0.072 ± 0.027 ^a^	0.106 ± 0.030 ^b^	0.172 ± 0.035 ^b^	0.067 ± 0.006 ^a^	0.132 ± 0.031 ^b^
C16:2 Δ9,11	0.167 ± 0.025 ^a^	0.179 ± 0.018 ^a^	0.239 ± 0.021 ^b^	0.276 ± 0.019 ^b^	0.164 ± 0.013 ^a^	0.184 ± 0.037 ^ab^
C18:2 Δ9,12	25.431 ± 1.733 ^a^	27.116 ± 1.818 ^b^	25.575 ± 1.317 ^a^	32.289 ± 2.704 ^c^	25.421 ± 0.668 ^a^	26.932 ± 2.962 ^ab^
C18:3 Δ6,9,12	0.239 ± 0.032 ^a^	0.259 ± 0.021 ^a^	0.718 ± 0.036 ^b^	1.165 ± 0.354 ^c^	0.248 ± 0.017 ^a^	0.307 ± 0.021 ^a^
Σ PUFA	26.170 ± 1.582 ^a^	28.662 ± 0.938 ^b^	26.030 ± 1.139 ^a^	32.190 ± 0.438 ^c^	25.431 ± 0.458 ^a^	28.414 ± 2.283 ^b^
AI	0.368 ± 0.043	0.369 ± 0.048	0.325 ± 0.021	0.399 ± 0.045	0.360 ± 0.027	0.362 ± 0.039
TI	0.526 ± 0.047	0.503 ± 0.055	0.424 ± 0.002	0.488 ± 0.047	0.497 ± 0.026	0.468 ± 0.027
HH	4.440 ± 0.676	4.516 ± 0.742	5.122 ± 0.135	4.055 ± 0.146	4.503 ± 0.376	4.753 ± 0.401
HPI	2.746 ± 0.342	2.743 ± 0.379	3.088 ± 0.201	2.531 ± 0.308	2.785 ± 0.218	2.783 ± 0.286

## Data Availability

Data are available on request.

## Supplementary Materials

The following supporting information can be downloaded at https://www.mdpi.com/article/10.3390/ijms27073201/s1.
