# Supplementary material for: Functionalization of Tenebrio molitor with Olive Mill Wastewater: Growth, Antioxidant Activity, and Metabolomic Insights
Source: Int J Mol Sci. 2026 Apr 1;27(7):3201. doi: 10.3390/ijms27073201 (PMC13073273; doi:10.3390/ijms27073201)
Supplement: Supplementary file 1 [file ijms-27-03201-s001.zip › ijms-4197519-supplementary.pdf]

Functionalization of *Tenebrio molitor* with Olive Mill Wastewater: Growth, Antioxidant Activity, and Metabolomic Insights

Annalaura Brai *, Giuseppe Galeone, Alessio Maccianti, Federica Poggialini, Chiara Vagaggini and Elena Dreassi

Department of Biotechnology, Chemistry and Pharmacy, University of Siena, via A. Moro-53100 Siena, Italy
Corresponding author: annalaura.brai@unisi.it, Via Aldo Moro 2, 53100, Siena, Italy

Contents

Table S1. Characterization of olive mill wastewater pH, TS, TSS, Dry matter, N and Fat. TPC and antioxidant activity (ABTS and DPPH). 3

Table S2. Average percentage composition of fatty acids in the analyzed olive mill wastewaters collected from the different olive mills. 3

Table S3. Nutritional composition of the reference diets as reported on product labels 4

Table S4. ^{1}H NMR assignments of selected metabolites in *Tenebrio molitor* larvae. 5

Figure S1. Score plots of Principal Component Analysis (PCA) 8

Figure S2. PCA Scree plot 9

Figure S3. PLSDA Classification Performance 10

Table S1. Characterization of olive mill wastewater pH, TS, TSS, N and Fat. TPC and antioxidant activity (ABTS and DPPH).

Sample	pH	TS (g/L)	TSS (g/L)	N (%)	Fat (%)	TPC (g AG eq/L)	ABTS (mmol Trolox /L)	DPPH (mmol Trolox /L)
		Mean ± SD	Mean ± SD	Mean ± SD	Mean ± SD	Mean ± SD	Mean ± SD	Mean ± SD
OWW1	6.18	75.82 ± 5.59[a]	5.26 ± 1.66[a]	2.21± 0.19[b]	2.62±0.27[c]	1.05±0.15[a]	18.34±0.18[a]	23.43± 0.91[a]
OWW2	5.01	101.08 ± 5.44[b]	10.85 ± 0.66[b]	2.21± 0.19[b]	0.59±0.02[a]	9.94±1.12[c]	104.39±5.56[b]	195.80± 8.87[b]
OWW3	5.24	107.95 ± 2.28[b]	9.90 ± 1.41[b]	0.11± 0.03[a]	1.11±0.06[b]	3.46±0.49[b]	163.96±5.44[c]	243.12± 15.62[c]

Total Solids (TS) and Total Suspended Solids (TSS) in the analyzed olive mill wastewaters (OWW) samples. Results represent the mean ± SD of three experiments. Values labeled with different letters (a, b, c, d) in the same column are significantly different ($p < 0.05$).

Table S2. Average percentage composition (± standard deviation, SD) of fatty acids (expressed as % of the total identified fatty acids) in the analyzed olive mill wastewaters (OWW) collected from the different olive mills.

FA	OWW1	OWW2	OWW3
	Mean ± SD	Mean ± SD	Mean ± SD
C14:0	0.06 ± 0.02	0.04 ± 0.01	0.04 ± 0.02
C16:0	12.99 ± 0.37[a]	12.07 ± 0.05[a]	13.07 ± 0.59[b]
C17:0	nd	0.21 ± 0.02	0.16 ± 0.03
C18:0	2.04 ± 0.08	2.06 ± 0.02	1.94 ± 0.19
C20:0	0.30 ± 0.02	0.32 ± 0.01	0.29 ± 0.04
Σ SFA	15.40 ± 0.30[b]	14.68 ± 0.05[a]	15.49 ± 0.76[b]
C16:1 Δ9	0.20 ± 0.05[a]	0.73 ± 0.01[b]	0.87 ± 0.00[b]
C16:1 Δ11	0.81 ± 0.02[b]	0.12 ± 0.01[a]	0.10 ± 0.01[a]

FA	OWW1	OWW2	OWW3
C17:1 Δ11	2.41 ± 0.43[b]	0.05 ± 0.01[a]	0.04 ± 0.01[a]
C18:1 Δ9	72.04 ± 0.89[a]	77.07 ± 0.14[b]	79.06 ± 3.92[c]
C20:1 Δ11	nd	0.29 ± 0.03	0.10 ± 0.10
Σ MUFA	76.45 ± 2.03[a]	78.25 ± 0.15[a]	80.16 ± 3.84[b]
C16:2 Δ9,11	0.06 ± 0.00	0.08 ± 0.00	0.08 ± 0.01
C18:2 Δ9,12	6.24 ± 0.15[b]	5.98 ± 0.48[a]	6.41 ± 0.01[b]
C18:3 Δ6,9,12	0.57 ± 0.01[a]	0.73 ± 0.09[b]	1.07 ± 0.08[c]
Σ PUFA	6.82 ± 0.16[a]	6.79 ± 0.57[a]	7.56 ± 0.10[b]

Results represent the mean ± SD of three experiments. Values labeled with different letters (a, b, c) in the same row are significantly different ($p < 0.05$). nd: not determined.

Table S3. Nutritional composition (%) of the reference diets as reported on product labels

Component	Wheat Bran (WB)	Oat Bran (OB)	Whole Oat Flakes (OF)
Energy (kJ)	1241	1481	1494
Protein	14	15	13
Fat	5.5	6.2	5.9
Saturated fat	0.9	1.2	1.0
Carbohydrates	27	52	58
Sugars	3.8	1.0	1.1
Fiber	42	15	10

Table S4. ^{1}H NMR assignments of selected metabolites in *Tenebrio molitor* larvae.

#	Metabolite	Chemical shift (ppm)	Type of proton
1	Isoleucine	0.94 (t)	δCH_3
		1.01 (d)	γCH_3
		1.25 (m)	γCH_2
		1.46 (m)	$\gamma^1 CH$
		1.98 (m)	βCH
		3.67 (d)	αCH
2	Leucine	0.96 (d)	δCH_3, $\delta^1 CH_3$
		1.72 (m)	βCH_2, γCH
		3.75 (m)	αCH
3	Valine	0.99 (d)	γCH_3
		1.05 (d)	$\gamma^1 CH_3$
		2.28 (m)	βCH
		3.61 (d)	αCH
4	Threonine	1.33 (d)	γCH_3
		3.59 (d)	αCH
		4.26 (m)	βCH
5	Alanine	1.48 (d)	βCH_3
		3.78 (q)	αCH
6	Arginine	1.68 (m)	γCH_2
		1.72 (m)	βCH_2
		1.89 (m)	δCH_2
		3.76 (dd)	αCH
		3.23 (t)	δCH_2
7	Glutamine	2.07 (dt)	βCH_2

#	Metabolite	Chemical shift (ppm)	Type of proton
		2.45 (m)	γCH_2
		3.77 (t)	αCH
8	Tyrosine	3.05 (dd)	βCH_2
		6.90 (d)	$H_{3,5}$ (ring)
		7.19 (d)	$H_{2,6}$ (ring)
		3.93 (dd)	αCH
9	Phenylalanine	3.14 (dd)	βCH_2
		7.32 (d)	$H_{2,6}$ (ring)
		7.37 (t)	$H_{3,5}$ (ring)
		3.98 (dd)	αCH
10	Tryptophan	3.25 (dd)	βCH_2
		7.20–7.65 (m)	indole H_2–H_7
		4.05 (dd)	αCH
11	Histidine	3.15 (dd)	βCH_2
		7.05 (s)	H_2 ring
		7.80 (s)	H_4 ring
		3.98 (dd)	αCH
12	Glucose α	5.23 (d)	H_1 (anomeric)
	Glucose β	4.65 (d)	H_1 (anomeric)
13	Trehalose	5.19 (d)	H_1,H_1' (anomeric)
14	Sucrose	5.41 (d)	Glc H_1
15	Acetic acid	1.92 (s)	CH_3
16	Citric acid	2.56 (dd)	CH_2
		2.70 (dd)	CH_2

#	Metabolite	Chemical shift (ppm)	Type of proton
17	Formic acid	8.46 (s)	CH
18	Fumaric acid	6.51 (s)	CH=CH
19	Lactic acid	1.33 (d)	CH_3
		4.11 (q)	CH
20	Succinic acid	2.40 (s)	CH_2
21	Glycerol	3.55–3.65 (m)	CH_2
		3.75 (m)	CH
22	Choline	3.19 (s)	$N(CH_3)_3$
		3.51 (m)	CH_2-N
		4.05 (m)	CH_2-O
23	Phosphocholine	3.23 (s)	$N(CH_3)_3$
		4.18 (m)	CH_2-O-P
24	Glycerophosphocholine	3.23 (s)	$N(CH_3)_3$
		3.50–4.30 (m)	glycerol protons
25	Betaine	3.27 (s)	$N(CH_3)_3$
		3.90 (s)	CH_2

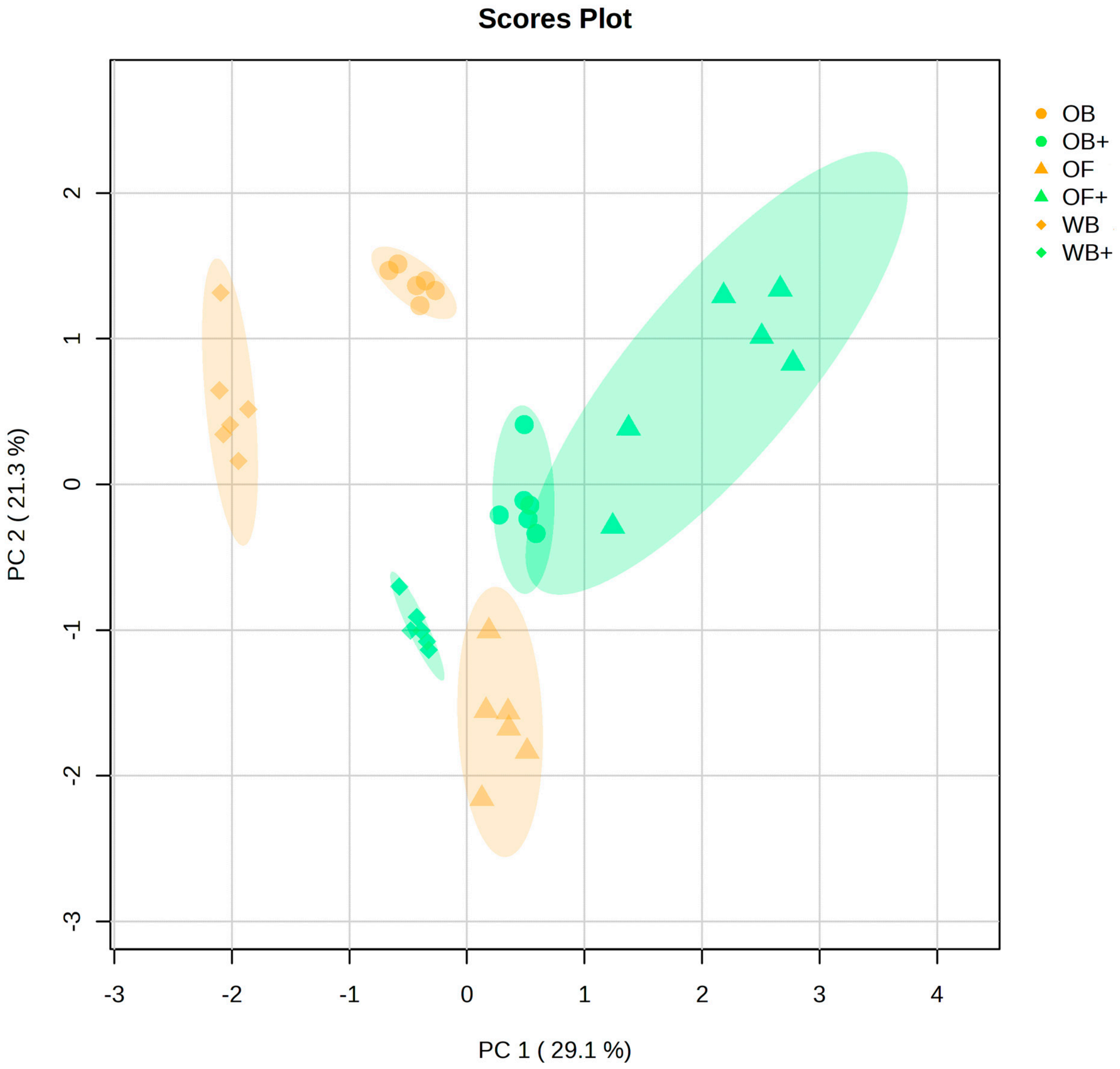

Figure S1. Score plots of Principal Component Analysis (PCA). *Tenebrio molitor* larvae fed with oat bran (OB), wheat bran (WB), whole oat flakes (OF) are reported in yellow, or diets supplemented with olive mill wastewater (green, OB+, WB+, OF+) after 45 days of rearing.

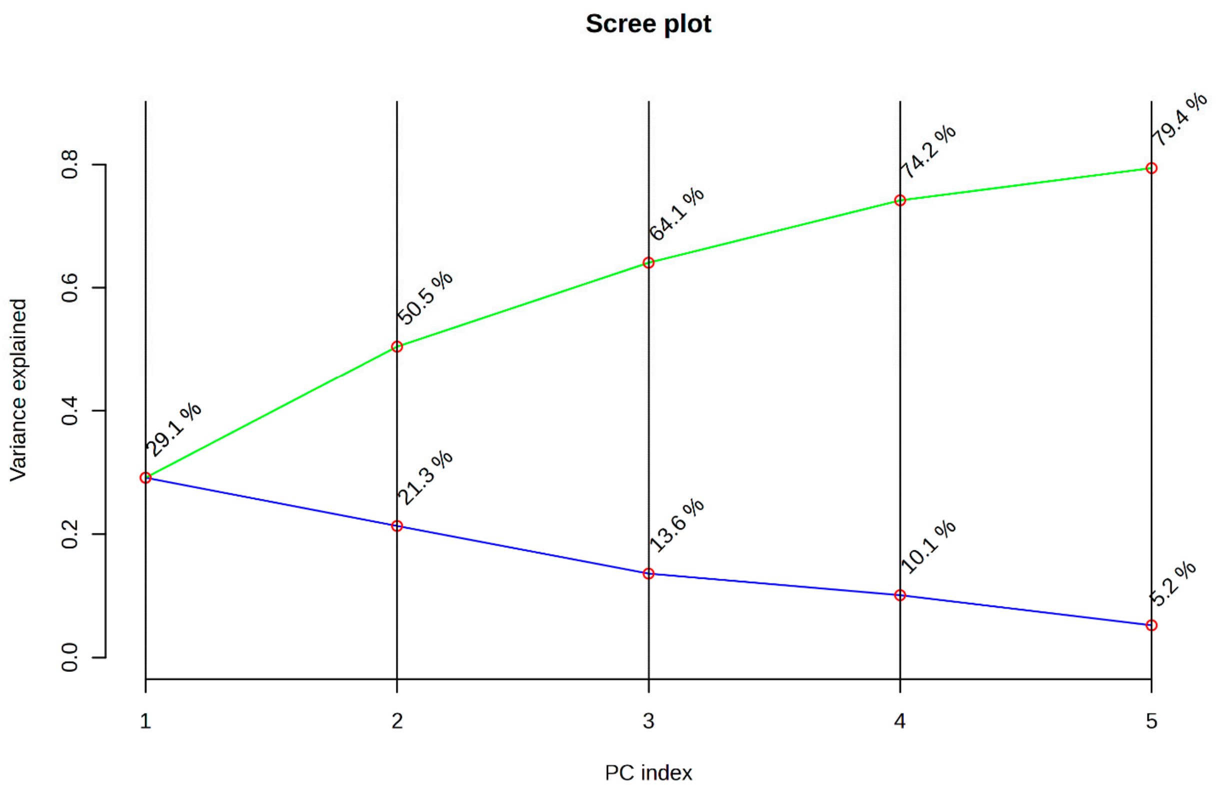

Figure S2. PCA Scree plot

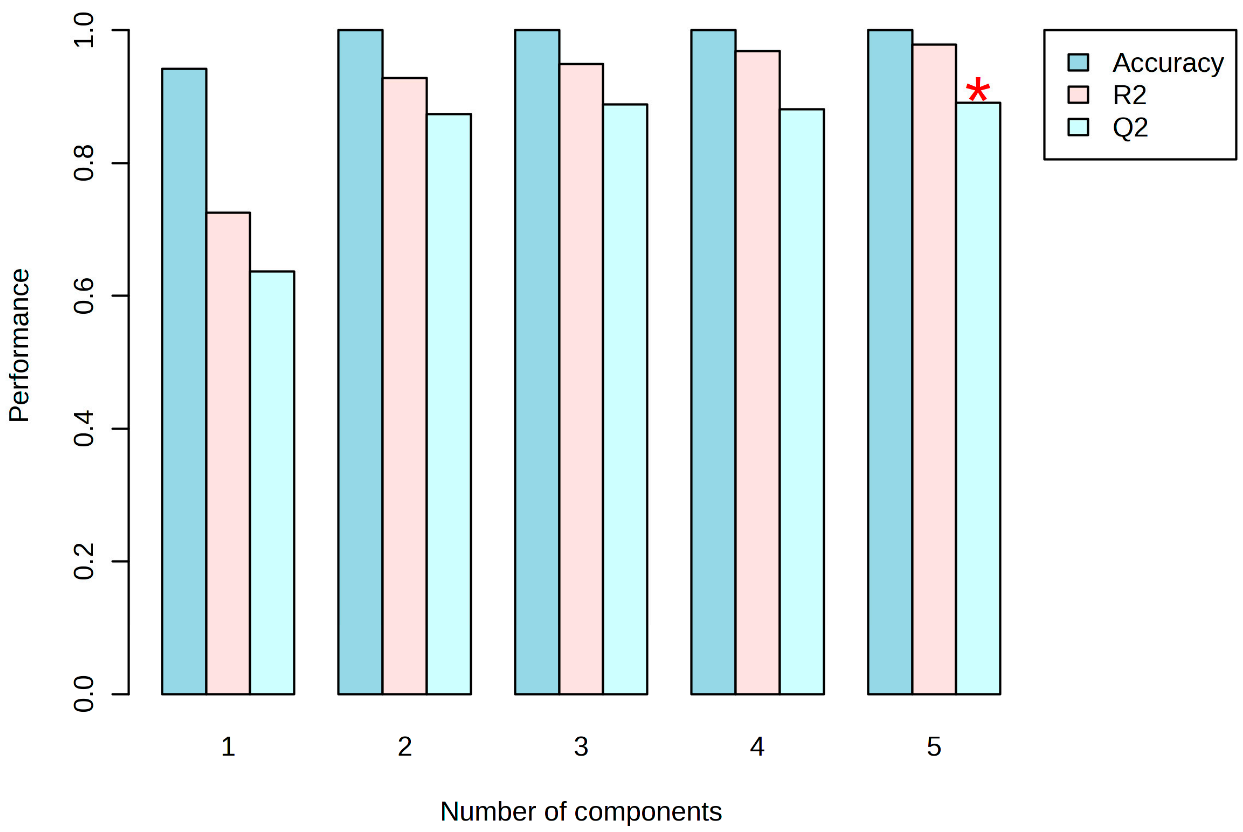

Figure S3. PLSDA Classification Performance
